# Факторы и состояния, влияющие на секрецию и функцию транскортина плазмы крови

**DOI:** 10.14341/probl13483

**Published:** 2025-07-22

**Authors:** А. Шевэ, Д. Г. Бельцевич, К. Ш. Гаджиева, Х. В. Багирова, А. К. Эбзеева, А. Н. Романова, М. М. Гаджимурадова, Г. А. Мельниченко

**Affiliations:** Национальный медицинский исследовательский центр эндокринологии им. академика И.И. Дедова; Национальный медицинский исследовательский центр эндокринологии им. академика И.И. Дедова; Национальный медицинский исследовательский центр эндокринологии им. академика И.И. Дедова; Национальный медицинский исследовательский центр эндокринологии им. академика И.И. Дедова; Национальный медицинский исследовательский центр эндокринологии им. академика И.И. Дедова; Национальный медицинский исследовательский центр эндокринологии им. академика И.И. Дедова; Национальный медицинский исследовательский центр эндокринологии им. академика И.И. Дедова; Национальный медицинский исследовательский центр эндокринологии им. академика И.И. Дедова

**Keywords:** транскортин, гормоны, связывающие белки, глюкокортикостероиды, буферная система, физиологические механизмы человека

## Abstract

Известно, что основная масса стероидных гормонов в крови находится в связанном с белками-переносчиками состоянии. Транскортин (кортикостероид-связывающий глобулин, SERPINA6), являясь основным транспортным белком стероидных гормонов, образуется главным образом в печени и регулирует системную биодоступность глюкокортикоидов и минералокортикоидов. В настоящей обзорной статье представлены данные литературы о влиянии различных факторов и состояний на ТК (синтез, секрецию и аффинность) и его роли в физиологических и патофизиологических процессах в организме. Снижение уровня ТК происходит в пожилом возрасте, при ожирении и метаболическом синдроме, на фоне таких заболеваний, как цирроз печени, сепсис, политравмы и обширные ожоги, а также при хирургических вмешательствах. Различные препараты могут оказывать влияние на концентрацию ТК в крови, так терапия эстрогенами (например, комбинированные оральные контрацептивы), вызывает значимое повышение ТК в крови, а назначение глюкокортикоидов, в свою очередь, приводит к снижению уровня ТК. Проведен в том числе анализ работ, направленных на изучение влияния ТК и изменение его уровня в крови при эндогенном гиперкортицизме.

## Abstract (en)

It is well-established that the majority of steroid hormones in the bloodstream are in a bound state, complexed with carrier proteins. Transcortin (corticosteroid-binding globulin, SERPINA6) serves as the principal transport protein for steroid hormones and is predominantly synthesized in the liver. Its primary function is to regulate the systemic bioavailability of glucocorticoids and mineralocorticoids. This review article presents an analysis of the existing literature on the impact of various factors and conditions on Transcortin, encompassing its synthesis, secretion, and affinity, as well as its role in physiological and pathophysiological processes within the human body. A reduction in Transcortin levels has been observed in several contexts, including old age, obesity, and metabolic syndrome. Additionally, this decrease is evident in the presence of cirrhosis, sepsis, polytrauma, extensive burns, and during surgical interventions. The concentration of Transcortin in the bloodstream can be influenced by a variety of medications. For instance, estrogen therapy, such as combined oral contraceptives, has been shown to induce a significant increase in Transcortin levels. Conversely, the administration of glucocorticoids has been associated with a decrease in Transcortin levels. Furthermore, this review article includes an analysis of studies that have investigated the influence of Transcortin and alterations in its blood levels in the context of endogenous hypercortisolism. These studies contribute to a deeper understanding of the complex interplay between Transcortin and steroid hormone regulation in various physiological and pathological conditions.

Транскортин (ТК, кортизол-связывающие глобулин, серпин А6) является основным транспортным белком глюкокортикостероидов (ГКС), в частности кортизола. Более 95% кортизола находится в связанном с белками-переносчиками состоянии, остальные 5% пребывают в свободном или активном состоянии и обладают способностью связываться со специфическими рецепторами в клетках. Основными белками — транспортерами кортизола являются ТК и альбумин. На долю ТК приходится до 80–90% гормона, он обладает высокой аффинностью, но низкой емкостью, в то время как альбумин имеет низкую аффинность и значительно большую буферную емкость и связывает до 10% кортизола [1].

ТК, который в основном вырабатывается гепатоцитами и в меньшей степени другими тканями, например желчным пузырем и почками, отвечает за своевременную и тканеспецифическую доставку кортизола к очагам воспаления, регулируя биодоступность кортизола для клеток-мишеней в нормальных и стрессовых условиях [2][3][4][5]. Более того, результаты исследования Gulfo и соавт. показали, что ТК синтезируется в надпочечниках. Отсутствие ТК приводит к снижению синтеза и секреции глюкокортикоидов в надпочечниках в ответ на стрессовые стимулы [6].

ТК был открыт Daughaday и соавт. и Bush и соавт. в 1956 году. Методом равновесного диализа было продемонстрировано присутствие в плазме крови специфических глобулинов, обладающих очень высоким сродством к кортикостероидам [7][8]. Впоследствии роль ТК в качестве переносчика была тщательно исследована. Накоплены данные о зависимости объема буферной системы и уровня циркулирующего кортизола. Настоящий обзор литературы представляет накопленные данные о различных факторах и состояниях, которые оказывают влияние на концентрацию и аффинность основного транспортного белка кортизола.

## ПОЛ И ВОЗРАСТ

Доказан половой диморфизм уровней ТК с его более высокими показателями у женщин [9]. Впервые о гендерных различиях в уровнях ТК сообщил De Moor и соавт. в 1967 г., когда при исследовании уровней ТК в популяции было зафиксировано бимодальное распределение значений ТК у женщин и его отсутствие у мужчин. Половые различия в уровне ТК могут быть связаны с более высоким уровнем эстрогенов у женщин, так как эстрогены являются хорошо известными индукторами секреции ТК через увеличение транскрипции генов и/или изменение гликозилирования молекулы ТК [10].

Физиологические изменения, возникающие в печени у пожилых людей с увеличением возраста, заключаются в уменьшении объема печени, печеночного кровотока, ее регенеративной способности, что приводит к снижению синтетической функции печени. Одновременно с уменьшением концентрации в крови альбумина, факторов свертывания крови и др. фиксируется более низкое содержание ТК в крови у пожилых людей, что подтверждается данными кросс-секционного анализа, в котором выявлена отрицательная корреляция между уровнем ТК и возрастом [9][11]. В исследовании Schlechte и соавт. выявлено, что связывающая способность ТК отрицательно коррелировала с возрастом, при этом не выявлено связи ни связывающей способности, ни аффинности ТК с концентрацией сывороточного белка в крови [12].

## ПОЛИМОРФИЗМ ГЕНА SERPINA6

Ген SERPINA6 включает в себя 5 экзонов и имеет длину в 19-kb и расположен на коротком плече 14 хромосомы (14q32.1). Промотор белка SERPINA6 содержит ТАТА-бокс и СААТ-бокс, а также другие элементы высококонсервативной последовательности ДНК, которые, по-видимому, обуславливают его специфическую печеночную экспрессию [13]. Ген экспрессируется в почках, легких, плаценте и поджелудочной железе [14][15].

В организме человека существуют полиморфизмы SERPINA6, которые снижают сродство ТК к кортизолу и влияют на его уровень в плазме [16][17][18][4]. Известно, что мыши с дефицитом ТК имеют более низкий уровень кортикостероидов и более высокий уровень свободных фракций и адренокортикотропного гормона (АКТГ), а также больший размер надпочечников [19][20]. В различных исследованиях выявлено до 20 полиморфизмов SERPINA6 человека, которые характеризовались дефектами в продукции или стероидосвязывающей активности ТК, и многие из них продемонстрировали воздействие на здоровье человека [21][4][22]. Наиболее распространенными симптомами у людей, имеющих мутации ТК, являются утомляемость, хроническая боль, гипотензия и, в некоторых случаях, избыточный вес или ожирение, хотя необходимо отметить, что не все люди с различными генетическими вариантами ТК имеют клинические признаки дефектов ТК [4]. В литературе описано четыре наследственных варианта ТК, ведущих к снижению сродства к кортизолу: Leuven L93H, Lyon D367N, G237V и Athens W371S. Наблюдается 3- и 4-кратное снижение аффинности кортизола при наличии ТК Leuven и Lyon соответственно [23][24]. Однако ни лабораторных, ни клинических изменений у пациентов с ТК Leuven не наблюдалось, а в свою очередь у пациентов с ТК Lyon D367N зафиксированы низкие показатели общего кортизола и ТК, при нормальном уровне АКТГ, свободного кортизола крови и мочи. При этом у пациентов клинически можно было наблюдать хроническую усталость, слабость, боль, депрессию, гипотонию и ожирение. Мутации SERPINA6 G237V и Athens W371S приводят к полной потере аффинности ТК к кортизолу [25][26]. В свою очередь Simard и соавт. идентифицировали несколько новых мутаций ТК человека, в различной степени влияющих на аффинность ТК к кортизолу (полностью отсутствовала — R260L, значительно снижена — H14Q, H89Y и умеренно снижена — I279F), способность связывать кортизол (H14R), синтез/секрецию (I48N и P246Q), чувствительность к протеолитическому расщеплению реактивного центра связывания (I179V и I279F) [22]. Несмотря на тот факт, что данные мутации встречаются редко, у пациентов с дефектным ТК может быть ошибочно диагностирован вторичный гипокортицизм [23–26].

В 2014 г. был создан консорциум под названием CORNET, основной задачей которого стало проведение метаанализа полногеномных ассоциаций (GWAMA). В 2023 г. Bankier и соавт. были представлены результаты анализа, основной целью которого было исследование генетических факторов, влияющих на вариабельность гипоталамо-гипофизарно-надпочечниковой оси (ГГНО) у 12 597 представителей европеоидной расы. Полученные данные свидетельствуют о том, что менее 1% вариаций уровня кортизола в крови связано с генетическими вариациями 14-й хромосомы. В исследуемом регионе были обнаружены различия в последовательностях ДНК в идентичных участках хромосом, заключающиеся в замене одного нуклеотида. Были выявлены три однонуклеотидных полиморфизма (SNP), которые ассоциированы, во-первых, с изменениями в способности ТК связывать кортизол, во-вторых, с вариабельностью концентрации общего ТК в крови, и, наконец, с изменением иммунологической реактивности петли активного центра ТК [27][28].

В гене SERPINA1 можно обнаружить два SNP, достоверно влияющих на уровень утреннего кортизола [29]. Первый SNP (SNP1) ассоциирован с более высоким уровнем утреннего кортизола, а также с увеличением общего количества молекул ТК в кровотоке. Согласно гипотезе «свободного гормона», общий уровень гормонов в плазме должен регулироваться для поддержания уровня свободных гормонов. Так, при наличии SNP1, большее количество молекул ТК приводит к снижению доступности кортизола, т.е. меньшее количество гормона будет свободно проникать в ткани, поэтому по принципу обратной отрицательной связи это приведет к стимуляции гипоталамо-гипофизарно-надпочечниковую ось (ГГНО). В результате чего компенсаторно происходит увеличение общего уровня кортизола. У субъектов с SNP1, несмотря на более высокий уровень общего кортизола, уровень свободного кортизола не отличался от уровня свободного кортизола, обнаруженного у субъектов с другими аллелями [27][28].

В свою очередь второй SNP (SNP2) в SERPINA1 ассоциирован с более низким утренним кортизолом, а также снижением сродства ТК. SNP2 представляют собой замену Leu115His, при котором снижается сродство ТК к кортизолу на 33%. Как и прежде, согласно гипотезе о «свободном гормоне», низкое сродство ТК к кортизолу будет приводить к уменьшению уровня связанного гормона, следовательно, больше гормона будет свободно проникать в ткани. Посредством обратной отрицательной связи будет происходить угнетение ГГНО, необходимое для снижения общего уровня кортизола до тех пор, пока уровень свободных форм не станут близкими к норме. Так, у субъектов с SNP2 общий уровень кортизола значительно ниже, чем у пациентов с ТК дикого типа, однако при измерении свободных фракций, те оставались неразличимы у двух групп исследуемых [27][28].

Третий SNP (SNP3) также встречается в гене SERPINA1, кодирующем альфа-1-антитрипсин, который может косвенно влиять на функцию ТК. Существует предположение, что SNP3 может изменить (возможно, увеличить) экспрессию альфа-1-антитрипсина. Это усилит подавление нейтрофильной эластазы, что приведет к увеличению связанного ТК кортизола и, соответственно, увеличению его буфера. Однако авторы отмечают, что эта ассоциация является самой слабой из трех и требует последующих дополнительных исследований [27][23][28].

## БЕРЕМЕННОСТЬ

Во время второго и третьего триместров беременности уровни ТК в материнской плазме увеличиваются в 2–3 раза, что сопровождается увеличением концентрации общего и свободного кортизола [30]. Повышение уровня ТК в плазме во время беременности связывают с эстроген-стимулируемым увеличением синтеза ТК в печени, а также удлинением его периода полувыведения [31]. Данный эффект достигается за счет специфического гликозилирования — модификации карбоксилазной группы ТК. Причина физиологического падения ТК плазмы и связанный с этим рост свободного кортизола на 38-й неделе беременности окончательно не изучены. Это явление может быть связано с усилением иммунного ответа (увеличение популяции Th1-Th2 Т-лимфоцитов). Рост уровня свободного кортизола плазмы, отмечаемый на поздних сроках беременности, свидетельствует об уменьшении отрицательной обратной связи кортизола на факторы, стимулирующие ГГНО; считается, что имеет место прямая (положительная) корреляция плацентарного гонадотропин-релизинг гормона (ГнРГ) и кортизола [32–34].

Кроме того, на поздних сроках беременности наблюдается значительное (12-кратное) увеличение концентрации прогестерона, в результате локальной продукции прогестерона плацентраными трофобластами, уровень стрероидов в межворсинчатом пространстве сильно отличается от их уровня в периферической крови матери и плода и вместе с тем увеличивается прогестерон/кортизол-молярное отношение [34]. Принимая во внимание тот факт, что человеческий ТК практически равносильно связывает прогестерон и кортизол, такое существенное увеличение прогестерона приводит к вытеснению кортизола из реактивного центра связывания ТК и, соответственно, приводит к изменению локальной концентрации и биодоступности глюкокортикостероидов (ГКС) трансплацентраного барьера [35]. В данном случае ТК выступает в качестве регулятора прогестерона во время беременности, уровень ТК в плазме коррелируют с циркулирующим прогестероном, а также с количеством прогестерона в межворсинчатом пространстве в первые два триместра [36]. При изучении исходов беременности и состояния здоровья новорожденных с дефицитом ТК у матерей никаких явных эффектов не наблюдалось.

Интересно, что среди детей, рожденных от ТК-дефицитных матерей, преобладали девочки, несмотря на то, что среди разных популяций при рождении обычно наблюдается небольшое преобладание мальчиков. Подобное явление наблюдалось после перенесенного сильного стресса во время беременности. Хотя биологическая причина искажения вторичного соотношения полов остается неясной, предполагается, что дефицит ТК может способствовать неадекватному воздействию кортизола или прогестерона на плод мужского пола, что может привести к его гибели. Эти данные могут указывать на избирательное преимущество эмбрионов женского пола в отношении выживания, поскольку они могут быть менее чувствительны к выраженным патологическим гормональным значениям. При этом необходимо учитывать, что, по неизвестным причинам, популяционная смертность выше у плодов и новорожденных мужского пола, о чем свидетельствуют половые различия в числе мертворождений и неонатальной смертности [36][37].

Кроме того, отмечено влияние физиологических изменений концентраций кортизола и ТК на изменения настроения на поздних сроках беременности и в послеродовом периоде. Послеродовой период может стать для матери новым и тяжелым, а изменение образа жизни может вызвать определенные изменения в настроении. Перепады настроения во время беременности обычно проходят через 10 дней после родов. Вопрос о том, как сохраняется длительная стабильность настроения, несмотря на постоянные сложные условия, остается неубедительным. В различных группах населения было показано, что снижение уровня кортизола связано с некоторыми психопатологиями, включая атипичную депрессию. Недавний метаанализ не выявил связи между гиперкортизолизмом и тяжелой депрессией в дородовом периоде, однако доказано, что гипокортизолизм связан с хроническими депрессивными состояниями [38][39]. Таким образом, определенный уровень кортизола может быть необходим для выработки определенных механизмов совладания и предотвращения некоторых аффективных расстройств. Аналогичным образом, исходя из данных работы Yavuz и соавт., физиологически высокий уровень кортизола на поздних сроках беременности может иметь конечное влияние на настроение матери [40].

## ТК ФЕТАЛЬНОГО И ПОСТНАТАЛЬНОГО ПЕРИОДА

Уровень ТК в амниотической жидкости человека на 24-й неделе беременности намного ниже, чем его уровень в сыворотке крови у небеременной женщины, и, по данным исследования Scott и соавт., составляет менее 300 нмоль/л (норма для взрослого человека — 367–810 нмоль/л) [41]. Фетальный ТК плазмы обычно начинает повышаться во втором триместре беременности, достигая максимума на поздних сроках (28–31 неделя беременности) с последующим постепенным снижением. Послеродовой, неонатальный уровень ТК остается низким в течение первой недели жизни, а затем постепенно поднимается, достигая нормы к 12-месячному возрасту [42].

ТК и ГКС в маточно-плацентарном и плодово-плацентарном кровообращении необходимы для органогенеза в период развития плода, в частности, для развития легких. Комплексы стероидов и ТК взаимодействуют со специфическими сайтами связывания на децидуальной оболочке эндометрия и синцитиотрофобластах плаценты. Плацентарный ТК состоит только из «трехантенных» форм олигосахаридов, характеризуется повышенным содержанием сиаловой кислоты, составляет 10% от ТК плазмы матери и имеет 60-кратно повышенную аффинность к клеточными мембранами синцитиотрофобласта, что указывает на роль для ТК в регуляции переноса стероидов и активности на границе между матерью и плодом [4][35][43][44][45].

Целью ретроспективного исследования Hodyl и соавт., включившим 351 новорожденных, было изучение взаимодействия прогестерона и кортизола с ТК во время развития плода. По результатам данной работы выявлено, что уникальным для плода образом очень высокие концентрации прогестерона (наблюдаемые на поздних сроках беременности) способны повышать концентрацию свободного кортизола за счет конкурентного связывания с ТК. Высокие концентрации циркулирующего фетального прогестерона конкурируют за связывание ТК с кортизолом, что приводит к 3-кратному увеличению фракции свободного кортизола в пуповинной крови. Более высокое соотношение свободного и связанного кортизола может изменять распределение кортизола в организме плода, способствуя выполнению кортизолом таких функций, как развитие центральной нервной системы и созревание легких [46].

## ОЖИРЕНИЕ, МЕТАБОЛИЧЕСКИЙ СИНДРОМ И ЭФФЕКТЫ НА СНИЖЕНИЕ ВЕСА

В значительном количестве исследований показано, что секреция кортизола увеличивается при ожирении, однако симультанно с этим ускоряется его метаболизм, и, таким образом, у части больных уровень циркулирующего кортизола остается нормальным [47][48]. В работе Marin и соавт. отмечена положительная корреляция между уровнем экскреции кортизола с мочой, окружностью талии (ОТ) и индексом талия/бедра (ИТБ), увеличение которых свидетельствует о накоплении висцерального жира у женщин с ожирением [49]. Pasquali и соавт. подтвердили, что суточная скорость экскреции кортизола была в 1,6 раза выше у женщин с абдоминальным распределением жира по сравнению с женщинами с гиноидным типом ожирения и контрольной группой женщин с нормальным весом [50]. В свою очередь в исследовании Fernandez-Real и соавт. обнаружена отрицательная корреляция между концентрациями ТК в плазме и показателями ожирения, включающими ИМТ, ОТ, ИТБ [9]. Причины изменений в ГГНО и уровней ТК при ожирении изучены лишь частично.

В большинстве случаев ожирение сопровождается развитием метаболического синдрома, характеризующегося преобладанием абдоминального типа ожирения, наличием гиперхолестеринемии с увеличением липопротеидов низкой плотности, гипертриглицеридемией, нарушением углеводного обмена (нарушение толерантности к глюкозе (НТГ) или сахарным диабетом 2 типа (СД2), и артериальной гипертензией (АГ). При метаболическом синдроме снижение ТК имеет многофакторную природу. У лиц с ожирением и резистентностью к инсулину (ИР) были получены достаточно противоречивые результаты с точки зрения уровня ТК. Согласно данным некоторых работ, у пациентов с ИР было зафиксировано снижение уровня ТК в плазме. В многочисленных исследованиях Fernandez-real и соавт. выявлено, что уровень ТК в плазме отрицательно коррелирует с уровнем инсулина в ходе орального глюкозотолерантного теста (ОГТТ) и положительно коррелирует с показателями глюкозы натощак, значением AUC глюкозы и гликированного гемоглобина (HbA1c). Вследствие этого было предположено, что концентрация ТК является маркером секреции инсулина. С другой стороны, низкий уровень ТК также может способствовать ИР за счет воздействия свободного кортизола на таргетные клетки, в том числе мышечные, что приведет к усилению метаболического действия кортизола и, соответственно, уменьшению захвата и утилизации глюкозы [9][51]. Однако в работах Holt и соавт. и Lewis и соавт. корреляций между уровнем инсулина и ТК не выявлено [52][53]. Несмотря на противоречивые результаты in vivo, в ходе исследований in vitro после введения инсулина наблюдалось дозозависимое снижение уровней мРНК ТК и секреции белка из клеток HepG2 [54].

С другой стороны, ТК является членом семейства SERPIN и имеет сайт связывания с эластазой, экспрессируемой на поверхности нейтрофилов, увеличение количества которых наблюдается как при ожирении, так и при ИР [55][56]. Следовательно, вариабельность циркулирующего ТК может быть связана с расщеплением ТК активированными нейтрофилами [2].

ИР и ожирение также сопряжены с хроническим воспалением, что сопровождается повышением С-реактивного белка, провоспалительных цитокинов — фактора некроза опухоли α (ФНО-α), интерлейкина-6 (IL-6), адипонектина [57][58]. Активация выработки ГКС происходит как вследствие ИР, так и через провоспалительные цитокины — IL-6 и ФНО-α [59][60].

## ХРОНИЧЕСКОЕ И ОСТРОЕ ВОСПАЛЕНИЕ

Существует тесное взаимодействие между иммунной и эндокринной системами, необходимое для поддержания и восстановления гомеостаза, в частности во время воспаления. При воздействии патогенов высвобождается большое количество цитокинов и других продуктов, которые, помимо своих собственных иммунных функций, могут также индуцировать нейроэндокринные реакции. После иммунного импульса цитокины (IL-1, IL-6), которые вырабатываются воспалительными клетками, стимулируют ГГНО, индуцируя высвобождение рилизинг-гормонов гипоталамуса, запуская цепную реакцию, что приводит к повышению концентрации ГКС. Кортизол, в свою очередь, регулирует уровень нескольких циркулирующих в крови провоспалительных цитокинов (IL-2, IL-3, IL-6), ФНО-α и интерферона-γ (ИФН-γ) и влияет на активность и жизнеспособность клеток иммунной системы, угнетает фагоцитоз антигенов и их последующую элиминацию макрофагами. Кортизол также оказывает угнетающее воздействие как на клеточный, так и на гуморальный иммунитет, что необходимо для поддержания баланса про- и противовоспалительных реакций [61]. Центр связывания IL-6 был идентифицирован в промоторе гена ТК [9]. Продолжительное подавление уровня ТК во время и после инфузии IL-6, наблюдаемое у здоровых испытуемых, наравне с подавляющим действием IL-6 на секрецию ТК клеточными линиями HepG2, могут служить доказательством того, что IL-6 является негативным регулятором ТК [62][63].

Хроническое воспаление коррелирует с измененной функцией ГГНО и формированием стойких или транзиторных нарушений в реализации биологических эффектов кортизола. Также оно часто связано с «относительным» гипокортицизмом, то есть с неадекватным или сниженным уровнем кортизола для данного воспалительного стимула. Этот феномен может быть связан с защитной адаптацией организма на стресс либо быть отражением нарушений на каком-либо уровне ГГНО [64][65]. Посттрансляционные изменения гликозилирования, обычно наблюдаемые в воспалительных гликопротеинах, генетические вариации или физиологическая модуляция активного центра связывания могут защитить ТК от расщепления, в частности нейтрофильной эластазой, особенно при хроническом воспалении [22][66–70]. Использование новой методологии твердофазного иммуноферментного анализа (ИФА), в ходе которого проводится количественный анализ нативной, связанной/высокоаффинной формы ТК (вТК) и расщепленной/свободной/низкоаффинной формы ТК (нТК) в сыворотке человека, позволило оценить расщепление ТК in vivo. В работах Lewis и соавт. и Nenke и соавт. на животных моделях зафиксированы одинаковые периоды полувыведения вТК и нТК. Одновременно с этим при хроническом воспалении в сыворотке крови наблюдались более высокие уровни нТК, что может косвенно указывать на изменение в буферизации кортизола при данном состоянии [71][72].

Существует мнение, что характеристики и роль ТК могут различаться у пациентов с хроническим и острым воспалением. Острое воспаление характеризуется опосредованной цитокинами активацией ГГН оси и резким снижением синтеза ТК, что приводит к транзиторному значительному повышению свободного кортизола крови [62][63][73]. Резкое снижение уровня ТК в крови ассоциировано с увеличением IL-6 и ГКС, также выявлено, что IL-1 действует на посттрансляционный процессинг и/или секрецию ТК в клетках печени. ТК широко изучался в условиях острого воспаления, при котором его уровень резко снижается, например, при ожогах, абдоминальных и кардиологических вмешательствах, остром панкреатите и сепсисе/септическом шоке [74-78]. При шоковых состояниях наблюдается острое истощение вТК, что может препятствовать адекватной доставке кортизола в ткани [79].

## CЕПСИС И ШОК

Общий уровень ТК значительно снижается при сепсисе и септическом шоке, в основе данного процесса лежит цитокин-опосредованное ингибирование печеночного синтеза белка и повышение уровня свободного кортизола [80]. В работе Nenke и соавт. представили результаты анализа корреляций общего ТК, вТК и нТК и тяжести течения, выживаемости у пациентов с сепсисом и септическим шоком. В ходе данного исследования было выявлено, что вТК значительно снижается при данных состояниях и его уровень пропорционален тяжести заболевания, так вТК снижается на 9% при сепсисе, на 51% у пациентов, переживших септический шок, и в 61% случаев у пациентов с летальным исходом по сравнению с группой контроля [79]. Падение концентрации вТК подтверждаются данными in vitro, свидетельствующими о том, что ТК расщепляется, превращая вТК в нТК при сепсисе. Также установлено, что доля циркулирующих нейтрофилов в общем количестве лейкоцитов коррелирует с увеличением доли нТК.

В более поздних исследованиях было подтверждено, что во время септического заболевания концентрация ТК продолжает прогрессивно падать, часто сопровождается с нарастающими полиорганными нарушениями [81]. После выздоровления уровень ТК в сыворотке возвращается к норме в течение нескольких дней [5]. У умерших пациентов после септического шока концентрация общего ТК в сыворотке крови была на треть ниже по сравнению с выжившими [82]. В последнем проспективном исследовании Meyer и соавт., в которое было включено 135 человек с септическим шоком, было показано, что у лиц с дефицитом ТК (<200 нмоль/л (референсный интервал 269–641 нмоль/л)), смертность в была в три раза выше по сравнению с теми, у кого этот уровень был выше 200 нмоль/л (отношение рисков равно 3,2) [5]. При многофакторном анализе низкая концентрация была одним из четырех независимых предикторов смертности; другими независимыми предикторами были оценка состояния здоровья по шкале Acute Physiology and Chronic Health Evaluation II (APACHE II) >25 баллов; шкала последовательной оценки органной недостаточности (SOFA) — оценка состояния печени, а также необходимость в заместительной почечной терапии [5]. Кроме того, отмечено, что дефицит ТК характерен только для заболеваний, связанных с сепсисом, и не наблюдается при критических состояниях, не связанных с ним [82].

При остром воспалении комбинированный эффект снижения кортизол-связывающей аффинности ТК, снижения синтеза ТК и повышенной секреции кортизола ожидаемо повысит уровень свободного кортизола, способствуя его благотворному плейотропному эффекту. Однако несмотря на обычно повышенный уровень свободного кортизола, только небольшая часть может достичь пораженных участков вследствие истощения вТК и потери резервуарной функции вТК. Более того, сохранение высокого уровня свободного кортизола в кровотоке, снижение уровня ТК и снижение метаболизма кортизола приводят к угнетению центрального контроля ГГН оси. Учитывая резкое падение вТК в группе летального исхода от септического шока, Nenke и соавт. предположили, что существует абсолютный уровень вТК, ниже которого кортизол не может эффективно выполнять свои противовоспалительные функции на клеточном уровне. Снижение вТК коррелирует с тяжестью заболевания в большей степени, чем общий или свободный кортизол, и, таким образом, авторами предложено использование вТК в качестве прогностического маркера при данных состояниях [79].

## ЭНДОГЕННЫЙ ГИПЕРКОРТИЦИЗМ И НАДПОЧЕЧНИКОВАЯ НЕДОСТАТОЧНОСТЬ

Существует ограниченное количество исследований уровня ТК крови у пациентов с эндогенным гиперкортицизмом центрального или надпочечникого генеза. В работе Schlechte и др. авторы выполнили анализ концентрации связывающей способности и аффиности ТК у 20 пациентов с эндогенным гиперкортицизмом преимущественно гипофизарного происхождения (16 человек), у одного исследуемого диагностирована кортикостерома надпочечника, у оставшихся — АКТГ-эктопированный синдром. ТК-связывающая способность снижалась на 40% по сравнению со здоровыми субъектами, более того, пациенты с гиперкортицизмом имели значительно более низкую концентрацию ТК, чем пациенты, получающие ГКС, и группа контроля. При этом связывающая способность не различалась в зависимости от локализации очага избыточной выработки кортизола. Не было также выявлено корреляции между уровнем суточной экскреции свободного кортизола с мочой или продолжительностью заболевания и ТК-связывающей способностью [12]. В более ранних работах Doe и соавт. и Murray и соавт. сообщают, что у 7 исследуемых пациентов с макронодулярной двусторонней гиперплазией надпочечников (МДГН) уровень и связывающая способность ТК оставались в пределах нормы, так как около 50% кортизола было в несвязанном состоянии [83–85]. В наблюдении Osorio и соавт. и De Moor и соавт. получены противоположные результаты в виде повышения ТК-связывающей способности [86][87]. В настоящее время накопленных данных недостаточно для формулировки определенных выводов. Работы, посвященные изучению ТК у больных с надпочечниковой недостаточностью, не проводились.

## ЛЕКАРСТВЕННЫЕ ВЕЩЕСТВА

## Эстроген-содержащие препараты

У здоровых женщин, использующих комбинированные оральные контрацептивы (КОК), содержащие низкую дозу эстрогена, наблюдается более высокая концентрация кортизола сыворотки и ТК. На данный момент нет утвержденных референсных интервалов для общего кортизола сыворотки, свободного кортизола в слюне и ТК для данной группы пациентов. Авторами Panton и соавт. предложены референсные интервалы с учетом результатов анализа показателей между женщинами, получающими и не получающими терапию КОК, которые составляют: 284–994 нмоль/л и 159–569 нмоль/л, а для ТК: 847–3366 нмоль/л и 860–1940 нмоль/л соответственно. Клинически значимых различий в уровне свободного кортизола слюны не обнаружено, в связи с чем авторами предложено использовать данный показатель для диагностики гиперкортицизма у женщин, принимающих эстрогены, поскольку кортизол в слюне не зависит от уровня эстрогена [88].

## Митотан

Митотан (1,1-дихлорфенилдихлорэтан) (о,п’-DDD) противоопухолевый препарат, который в настоящее время является безальтернативным и эффективным препаратом для лечения адренокортикального рака (АКР). Митотан оказывает специфическое прямое цитотоксическое действие на митохондрии коры надпочечников, нарушая стероидогенез. Данный препарат оказывает эстроген-подобное действие, увеличивая синтез ТК в печени. Результаты исследования Nader и соавт. подтвердили, что у пациентов, получающих лечение митотаном в дозе 4–6,5 г в день, резко повышается уровень ТК. Также было зафиксировано, что при воздействии митотана на клетки HepG2, сохранялась синтетическая способность клетки. В дозах, эквивалентных рекомендуемым терапевтическим концентрациям, о,п-ДДД значительно стимулировал секрецию ТК с увеличением экспрессии эндогенной мРНК гена SERPINA6 [89]. В недавнем исследовании также продемонстрировано, что пациенты с АКР и терапевтической концентрацией митотана в плазме крови имели более высокую концентрацию ТК в сыворотке крови по сравнению с пациентами с новообразованиями надпочечников или беременными женщинами (группа контроля), а также более высокий уровень кортизола в сыворотке крови [90].

## Глюкокортикостероиды

ГКС регулируют уровень своего транспортного белка ТК посредством обратной отрицательной связи [3]. У людей уровень ТК в плазме снижается при длительном воздействии ГКС как эндогенного (при синдроме Кушинга (СК)) [19], так и экзогенного происхождения (при введении синтетических ГКС) [91].

По результатам работы Pugeat и соавт. преднизолон обладает сродством к ТК практически равном кортизолу. Однако данный белок имеет очень низкое сродство к большинству других синтетических ГКС, таких как дексаметазон [91]. У пациентов, получавших ГКС, связывающая способность была значительно ниже, чем в группе контроля, однако их аффинность была одинаковой. Более того, пациенты, получавшие лечение фармакологическими дозами ГКС, имели снижение связывающей способности на 24–30%, в то время как получавшие заместительную терапию ацетатом кортизона или преднизолоном имели незначительное отклонение данного показателя. У пациентов, получавших дексаметазон, связывающая способность существенно не различалась с группой контроля [12].

## ЗАКЛЮЧЕНИЕ

В настоящий момент требуется накопление дальнейших данных о клинической значимости исследования уровня ТК в практике. Несмотря на достаточно значительное количество данных об основном транспортном белке кортикостероидов (обобщенные данные представлены в таблице 1), ТК, на сегодняшний день вопрос о его значении и влиянии на течение различных заболеваний, сопровождающихся СК, остается открытым. Многообещающим является проведение дополнительных исследований о роли ТК и других факторов, способных в том числе оказывать влияние на результаты гормонального обследования. В частности, перспективными представляются исследования концентрации и изменения свойств ТК у больных с функционально-автономной продукцией кортизола, а именно при МДГН, с целью изучения патогенеза патофизиологического ответа организма на длительную умеренную гиперкортизолемию, наблюдаемую при данной патологии. Возможно, что данный белок может оказывать непосредственное влияние на достижение и сохранение долгой ремиссии после перенесенного одностороннего оперативного вмешательства. Необходимы дополнительные исследования по изучению возможности использования ТК как предиктора развития рецидива гиперкортицизма при МДГН.

**Table:** Таблица 1. Лекарственные препараты и состояния, которые могут оказывать влияние на уровни ТК

Условия	ТК	Механизм	Источники
Гормоны и медикаменты
Эстрогены (в том числе комбинированные оральные контрацептивы)	↑↑↑ (>50%)	Синтез и/или секреция	[88]
Митотан	↑	Синтез и/или секреция	[89][90]
IL-1β	↑		[62]
Глюкокортикоиды -плод -взрослые	↑ ↓	Синтез и/или секреция	[12][91]
Инсулин	↓	Синтез и/или секреция	[9][51][54]
ИФР-1	↓	Синтез и/или секреция	[54]
IL-6	↓	Синтез и/или секреция	[62][63][74]
Состояния
Возраст >65 лет	↓	Синтез и/или секреция, выведение	[9][11][12]
Беременность	↑	Синтез и/или секреция, выведение	[30][31]
Синдром Кушинга - Надпочечного генеза - Гипофизарного генеза - АКТГ-эктопия	N ↓ ↓	Синтез и/или секреция	[83–85] [12] [83][85]
Ожирение	↓	Синтез и/или секреция	[9][49][50]
Дефицит массы тела	↓	Синтез и/или секреция	[92][93]
Гестационный диабет	↓	Синтез и/или секреция	[94]
Цирроз печени	↓	Синтез и/или секреция	[83]
Острый панкреатит	↓	Синтез и/или секреция	[77]
Сепсис, септический шок	↓↓↓ (>50%)	Синтез и/или секреция	[78][79]
Ожоговая травма	↓↓	Синтез и/или секреция	[74]
Множественная травма	↓↓	Синтез и/или секреция	[78]
Хирургическое вмешательство	↓↓	Синтез и/или секреция	[75][76]

Хотя мы добились прогресса в понимании того, как регулируются уровни плазменного и тканевого ТК во время воспаления, наши знания о его роли и воздействии ограничены. Это подчеркивает необходимость проведения дополнительных исследований. Остается открытым вопрос о роли ТК в процессе восстановления, равно как и время нормализации уровней ТК в плазме при различных состояниях.

Представляется интересным также дальнейшее исследование полиморфизмов SERPINA6 и изучение эффектов этих вариантов ТК как в нормальных физиологических состояниях, так и патологических. Выявление пациентов с дефицитом ТК является трудной диагностической проблемой, так как нарушения производства и/или функции ТК не всегда связаны с неспецифическими признаками надпочечниковой недостаточности.

## ДОПОЛНИТЕЛЬНАЯ ИНФОРМАЦИЯ

Источники финансирования. Работа выполнена в рамках государственного задания «Разработка новых технологий диагностики и мониторинга опухолей коры надпочечников с использованием метаболомных и протеомных технологий». Регистрационный номер 123021300098-7.

Конфликт интересов. Конфликт интересов отсутствует.

Участие авторов. Все авторы одобрили финальную версию статьи перед публикацией, выразили согласие нести ответственность за все аспекты работы, подразумевающую надлежащее изучение и решение вопросов, связанных с точностью или добросовестностью любой части работы.
